# A Comparative Study on *Trichoderma harzianum* and a Combination of *Candida*/*Bacillus* as Tools for the Bioremediation of Table Olive Processing Water

**DOI:** 10.3390/microorganisms8060878

**Published:** 2020-06-10

**Authors:** Daniela Campaniello, Antonia Carlucci, Barbara Speranza, Maria Luisa Raimondo, Francesca Cibelli, Maria Rosaria Corbo, Antonio Bevilacqua

**Affiliations:** Department of the Science of Agriculture, Food and Environment (SAFE), University of Foggia, 71122 Foggia, Italy; daniela.campaniello@unifg.it (D.C.); antonia.carlucci@unifg.it (A.C.); barbara.speranza@unifg.it (B.S.); marialuisa.raimondo@unifg.it (M.L.R.); francesca.cibelli@unifg.it (F.C.)

**Keywords:** pollution, table olives, washing, debittering, fungi, bacteria, bioremediation

## Abstract

A comparative study was performed on *Trichoderma harzianum* and a combination of *Candida boidinii*/*Bacillus pumilus* to reduce the polluting effect of TOPW (Table Olive Processing Water) from the Spanish style. A 2^k^ fractional design was used to study the effect of pH (6–11 for the fungus and 6–9 for *Candida*/*Bacillus*), temperature (10–35 °C) and duration (7–14 days for *Candida*/*Bacillus* and 14–21 days for *T. harzianum*), and the effect on phenol reduction, COD and color was evaluated. The experiments were also performed on diluted TOPW (dilution ratio 1:1). Generally, *Trichoderma* removed higher amounts of phenols and reduced COD more than the combination *Candida*/*Bacillus*, thus confirming the higher efficiency of filamentous fungi reported in the literature. The dilution of TOPW had an effect only on COD reduction; however, the effect was mild, at least for *T. harzianum* (4%), while yield increase was 9% for *Bacillus*/*Candida*. pH acted in a different way on phenol removal and COD reduction; an increase of pH caused a reduction of efficiency for COD, while the effect was positive for phenols.

## 1. Introduction

Table olive production is a predominant economic activity of Mediterranean countries. In recent years, the average annual world production was around 2.6 million tons, and European countries contributed 30% of the total production, in particular Spain, Greece and Italy with 72, 16 and 9% of the total EU production, respectively. Egypt, Turkey, Algeria, Syria and Morocco are high producers, with a total production of 1,223,500 tons (season 2015/2016). The United States and some South American countries (Argentina, Mexico and Peru) are also important producers, and contribute 70,500 and 151,500 tons (season 2015/2016), respectively [1,2].

Due to the high production of table olives, industries produce a high quantity of wastewaters (including processing water used for washing after debittering; Table Olive Processing Wastewater, TOPW) with many problems of management and environmental pollution; the reuse of these industrial waters, for example for agriculture, would solve a major problem of water shortage, in addition to directly providing nutrients to the soil [3]. TOPW consists of water from the washing of olives after debittering; it is greyish-to-orange in colour, mostly containing sand or grains of soil. It is also composed of organic pollution (expressed as Biological and Chemical Oxygen Demand, BOD and COD, respectively) and phenol compounds resulting from the hydrolysis of oleuropein (when olives are treated with sodium hydroxide).

The volumes and compositions of TOPW rely on the methods applied; Californian- and Spanish-styles involve lye treatment, and repeated washings for the removal of alkalinity; thus, they are considered the most polluting styles. In detail, the production of Californian-style black-ripe olives causes the highest pollutant potential (a maximum total volume of ∼6 L/kg olives produced) [1].

Nowadays, the disposal of TOPW is approached with physical and chemical methods with moderate success [4]. Ozonation, UV irradiation, photocatalysis, hydrogen peroxide oxidation, Fenton’s reaction, electrochemical oxidation and wet air oxidation are adequate methods to reduce the organic matter content, but their costs are still too high [2].

Alternatively, biological treatments meet the current necessity of disposing of these by-products through an environmentally friendly green economy, which uses microorganisms to remove organic matter in wastewaters.

Due to their phenol degrading ability, many bacteria (*Bacillus*, *Pediococcus*, *Lactobacillus*, *Arthrobacter*, *Azotobacter*, *Pseudomonas*, *Ralstonia*) and fungi (*Phanerodontia chrysosporium*, *Trametes versicolor*, *Trametes trogii*, *Lentinus edodes*, *Aspergillus niger* and *A. terreus*), are used for the detoxification of olive oil mill wastewaters (OMWW) [5,6,7,8]. On the other hand, few data are available on the bioremediation of TOPW. Therefore, the main goal of this research was to design a bioremediation process of TOPW from green olive production through fungi and bacteria.

The specific objectives were: (a) to assess the effect of microorganisms on the residual content of phenols; (b) to evaluate their ability to reduce COD; (c) to point out the impact of some processing variables (pH, temperature and duration) on the effectiveness of the treatment; (d) to evaluate the effect of TOPW dilution (a common practice of Southern Italy). *Trichoderma harzianum* and a combination of *Candida boidinii*/*Bacillus pumilus* were used as biological tools.

## 2. Materials and Methods

### 2.1. Microorganisms

*B. pumilus* (formerly known as *Bacillus* sp. strain 13M) [7], *C. boidinii* (strain 682) [8] and *T. harzianum*, strain Var119 [9,10], were used throughout this study. The bacterium was maintained on Tryptone Soya Agar (Oxoid, Basingstoke, UK) at 4 °C and grown in Tryptone Soya broth at 30 °C for 48 h; the yeast was maintained on YPG agar (yeast extract, 10 g/L; peptone, 20 g/L; glucose, 20 g/L) and grown in YPG broth incubated at 25 °C for 72 h, while the fungus was maintained on PDA (potato, 200 g/L; dextrose, 20 g/L; agar 20 g/L) at 4 ± 3 °C, and grown on PDA at 23 ± 2 °C for 15 days in the dark.

### 2.2. Processing Water

TOPW was supplied by a factory located in Cerignola (Foggia county, Apulia); it was collected in October (season 2015) from olives processed through the Spanish style.

### 2.3. Bioremediation through Bacillus pumilus and Candida boidinii

The strains were harvested by centrifugation (1000× *g* for 10 min) and used to inoculate TOPW to 6 log CFU/mL for each microorganism. Immediately before inoculation, the pH of TOPW was adjusted to 6.0 or 9.0 through HCl 1.0 N; the samples were stored at 10 and 35 °C for 7 or 14 days.

Storage temperature (10 or 35 °C; that is, cardinal values for *B. pumilus*), pH (6.0 or 9.0; the optimal and maximum value for *B. pumilus* growth) and the duration of the experiments (7 or 14 days, chosen because preliminary experiments revealed that, after 14 days, the bacterium experienced a strong viability loss) were combined through a full 2^k^ design. The combinations are in Table 1.

A second experiment was peformed by diluting TOPW with sterile tap water (dilution ratio 1:1).

The samples were analyzed immediately after the inoculation, and after 7 and 14 days, to assess the following parameters: pH, colour, the viable count of bacterium and yeast, phenol content and COD. Uninoculated samples were used as controls.

### 2.4. Bioremediation through Trichoderma harzianum

*T. harzianum* propagules (6 log CFU/mL) were inoculated in TOPW. Immediately before inoculation, the pH was adjusted to 6.0 through HCl 1.0 N (the initial pH was 11.0); after inoculation, the samples were stored at 10 and 35 °C for 14 or 21 days. Uninoculated TOPW was used as control.

Temperature (10 or 35 °C; that is, the same range of the combination yeast/bacterium), pH (6.0 or 11.0; that is, a pH around neutrality and the value usually found in TOPW), and the duration of the experiments (14 or 21 days; optimal for the growth of the fungus) were combined through a 2^k^ the design (Table 2). A second experiment was performed by diluting processing water with sterile tap water (dilution ratio 1:1).

The samples were analyzed immediately after the inoculation, and after 14 and 21 days, to evaluate pH, colour, fungal biomass, phenol content and COD.

### 2.5. Analyses

The pH was evaluated with a pH-meter Crison mod. 2001 (Crison instruments, Barcelona, Spain), while the colour was evaluated with a colorimeter Minolta (Konica Minolta Europe, Milan, Italy).

The viable count was evaluated by spreading on Tryptone Soya Agar + 0.17 g of cycloheximide incubated at 30 °C for 24–48 h (*B. pumilus*), and on YPG agar + 0.1 g of chloramphenicol, incubated at 25 °C for 48–72 h (yeast). The counts were confirmed by microscopic examination.

For the fungal biomass, all liquid cultures were filtered through sterile Buckner funnels (70 mm diameter) in order to retain the mycelium on Miracloth paper (Calbiochem, CA) previously conditioned in a stove at 105 °C for 24 h. The weight of each fungal mycelium was determined after drying in the stove at 105 °C.

Phenols were determined using the method of Folin–Ciocalteu. Gallic acid (Sigma-Aldrich, Milan, Italy) was used as standard.

Chemical Oxygen Demand (COD) was assessed using a standardized COD cuvette test (15–150, 150–1000, and 1000–10,000 mg/L O_2_) from Hach Lange (HACH LANGE GMBH, Düsseldorf, Germany).

### 2.6. Design of Experiment Analysis

The experiments were performed on at least two independent samples (TOPW collected in two different periods) and repeated twice for each batch.

The reduction of phenols and COD throughout storage was used as the input for a DoE analysis (Design of Experiments) through the software Statistica for Windows, ver. 12.0 (Statsoft, Tulsa, OK, USA); pH, temperature and duration were used as categorical predictors (or independent variables).

The model was built using the option ‘2-way interaction’, for the evaluation of individual (‘pH’, ‘temperature’, ‘duration’) and interactive effects (‘pH × temperature’, ‘duration × temperature’, ‘pH × duration’). The significance of the model was evaluated through the adjusted regression coefficients and the mean square residual, whereas the significance of each factor was assessed through a Fisher test (*p* < 0.05). A second output of the polynomial equation is the 3D plot.

### 2.7. Prediction Profiles

The effect of temperature and pH on the dependent variables (COD and phenol reduction) was evaluated through the prediction profiles, showing the values assumed by the dependent variables as a function of each predictor, while the other two independent variables were set to constant values.

The following conditions were used: (i) pH profile, temperature at 22.5 °C and duration at 14 days; (ii) temperature profile, pH at 8.5 and duration at 14 days.

### 2.8. Multivariate ANOVA

COD and phenol amounts were modelled as decreasing (%):

(1) Removal=[(B−E)/B]*100

where B is the value of COD or phenols before inoculation, and E is the value after 7, 14 or 21 days. These values were used as the input to run a MANOVA (multivariate analysis of variance), with the duration of the experiment, pH, temperature and dilution being the categorical predictors; Tukey’s test was used as the post-hoc comparison test (*p* < 0.05).

## 3. Results

### 3.1. Bacllus pumilus/Candida boidinii

Table 3 shows the effects of temperature, duration and pH on COD reduction by the combination *B. pumilus*–*C. boidinii* for undiluted TOPW. The reduction of COD was mainly affected by the linear terms of temperature (as the positive term) and duration (as the negative term); all other effects were not significant.

**Figure 1:**
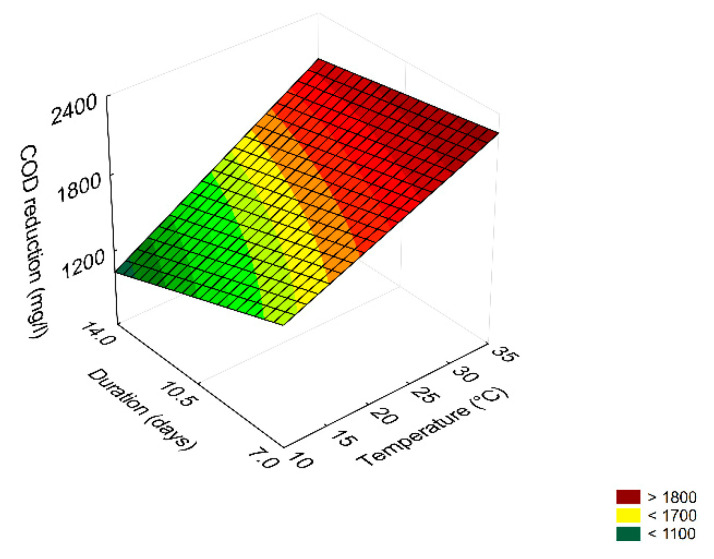
Surface response plot for the interaction duration/temperature on COD reduction by the combination of *B. pumilus*–*C. boidinii* strains.

The table of effects is a qualitative output; that is, it can highlight if a term is significant or not but it cannot point out the extent to which a term is significant; this information can be easily obtained through 3D-plots. As an example, Figure 1 shows the interaction ‘duration by temperature’ on COD reduction. This trend showed the best performance, that is, the highest reduction (ca. 2400 mg/L), at 35 °C and after 7 days. Otherwise, the minimum value of COD reduction (<1100 mg/L) was found after 14 days at 10 °C.

**Figure 2:**
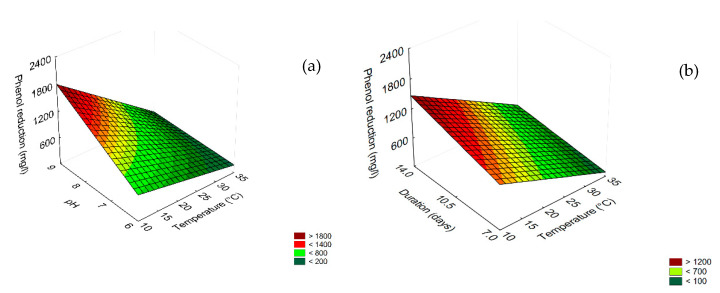
Surface response plots for the interaction pH/temperature (**a**), duration/temperature (**b**) on phenol reduction by the combination of *B. pumilus*–*C. boidinii* strains.

Concerning phenol reduction (Table 3), the most significant term was temperature (as a negative linear term), followed, in descending order, by pH (as a linear term), the interaction [Temperature] × [pH] (as negative term), and by the linear term of duration. The highest phenol reduction (>1800 mg/L) was obtained at pH 9/10 °C (Figure 2a), or after 14 days (>1200 mg/L) (Figure 2b).

**Figure 3:**
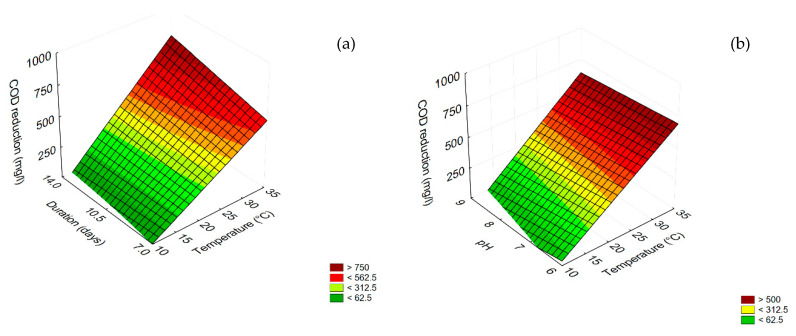
Surface response plots for the interaction temperature/duration (**a**) and temperature/pH (**b**) on COD reduction in diluted TOPW by the combination of *B. pumilus* – *C. boidinii* strains.

The same approach was performed on diluted TOPW. For COD reduction, all independent factors played a significant role, but temperature was the most important (Table 3). The quantitative trend is shown in Figure 3; the highest COD reduction (800 mg/L) was found at 35 °C and after 14 days (Figure 3a). The effect of pH was less significant, because the differences between pH 6 and 9 were less pronounced (COD reduction 650 mg/L at pH 9 and 700–730 mg/L at pH 6.0) (Figure 3b).

### 3.2. Trichoderma harzianum

**Figure 4:**
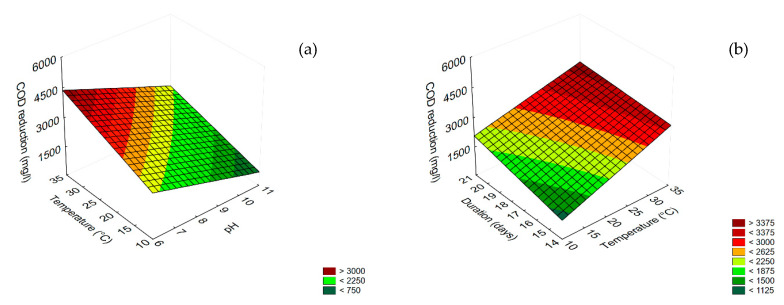
Surface response plots for the interaction temperature/pH (**a**) and temperature/duration (**b**) duration/pH (C) on COD reduction by *T. harzianum*.

**Figure 5:**
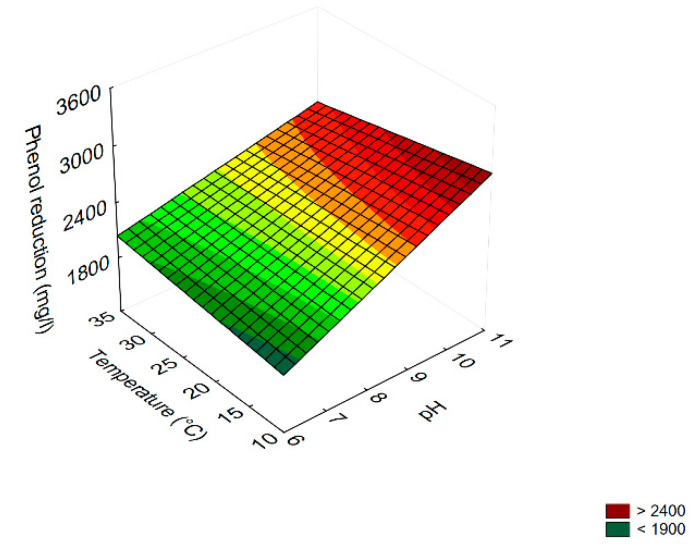
Surface response plot for the interaction Temperature/pH on phenol reduction by *T. harzianum*.

The results concerning COD and phenol reduction for undiluted TOPW are shown in Figure 4 and Figure 5. Concerning COD reduction, all variables were significant as linear and interactive terms, except for the interaction [pH] × [Temperature] (Table 3), but the best performance (COD reduction ca. 4000 mg/L) was found for the combination pH 6/35 °C (Figure 4a); time (the duration of the experiment) played a positive role, as COD reduction increased as time increased, and the highest value was found after 21 days (>4000 mg/L) (Figure 4b).

The most significant term for phenol reduction was pH, followed by the interaction [pH] × [temperature] (as negative term) (Table 3). The correlation of these variables with phenol reduction is in Figure 5, which shows that the performance (phenol reduction) was increased by increasing the pH and reducing the temperature, with a maximum (phenol reduction > 2800 mg/L) at pH 11/10 °C.

**Figure 6:**
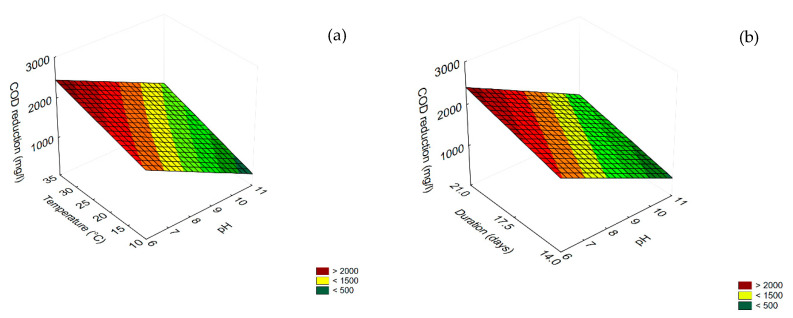
Surface response plots for the interactions temperature/pH (**a**) and duration/pH (**b**) on COD reduction in diluted TOPW by *T. harzianum*.

**Figure 7:**
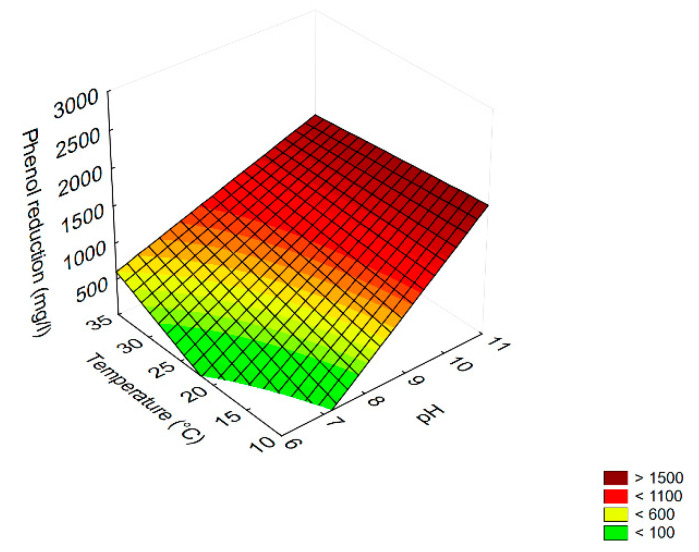
Surface response plot for the interaction temperature/pH on phenol reduction in diluted TOPW by *T. harzianum*.

The results for the effect of *T. harzianum* on diluted TOPW are in Figure 6 and Figure 7. For COD reduction, all variables played a significant role as linear terms, although the pH was the most significant (Table 3). Figure 6a,b show the surface response plots for the interactions ‘temperature by pH’ and ‘duration by pH’, respectively. A COD reduction of ca. 2500 mg/L was achieved at the highest temperature (35 °C) and pH 6 (Figure 6a) or after 21 days (Figure 6b).

For phenol reduction, all variables were significant as linear and interactive terms, but the most significant was pH, followed by duration and [pH] × [temperature] (Table 3). The highest phenol reduction (>1500 mg/L) was obtained at the highest pH (10–11) independently of temperature (Figure 7).

### 3.3. Effect on the Colour and on the Biomass

Generally, *T. harzianum* and the combination *Bacillus/Candida* caused a strong reduction of b-parameter (yellow/brownish colour) by 50–60% or more, but the effect of factors was not significant (data not shown). The biomass of *T. harzianum* was from 60 to 200 mg, while bacterial and yeast strains were at 5–6 log CFU/mL after 14 days.

### 3.4. Prediction

The second step of this research was aimed at building prediction profiles for pH and temperature to compare the performances of *Bacillus/Candida* and *T. harzianum*. A prediction profile does not show actual values, but predicted ones. It is generally built for each predictor per time, while the others are set to constant values.

**Figure 8:**
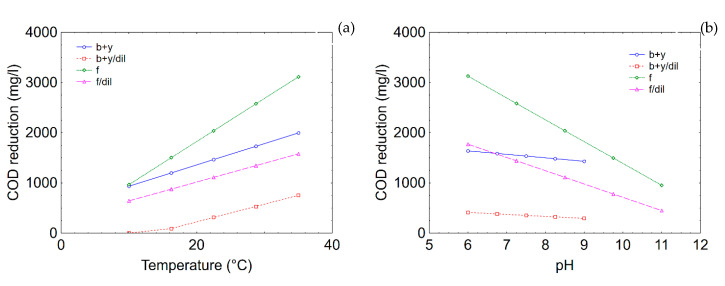
Prediction profiles for the effects of temperature (**a**) and pH (**b**) on COD reduction. b + y, combination *Bacillus/Candida*; f, fungus (*T. harzianum*); dil, diluted TOPW.

In this research, the values were set to 22.5 °C (temperature), 8.5 (pH) and 14 days (duration), because they are all included in the ranges used for both *Bacillus/Candida* and *T. harzianum*. Figure 8 shows the prediction profiles for COD reduction. Temperature played a unique role, because the model predicted an increase of the performance as a function of temperature; that is, from 933 to 1997 mg/L for *Bacillus/Candida* and from 966 to 3115 mg/L for *T. harzianum* in the case of undiluted TOPW (Figure 8a). As expected, the extent of COD reduction was lower in diluted TOPW.

The correlation pH/COD reduction was negative, because an increase of pH caused the reduction of the performances; however, the effect of pH was stronger on *T. harzianum,* with a decrease of COD reduction in processing water from 3128 to 953 mg/L (from pH 6 to 11), while the decrease was less significant for the combination *Bacillus/Candida* (COD reduction was 1640 mg/L at pH 6.0 and 1430 mg/L at pH 9.0) (Figure 8b).

**Figure 9:**
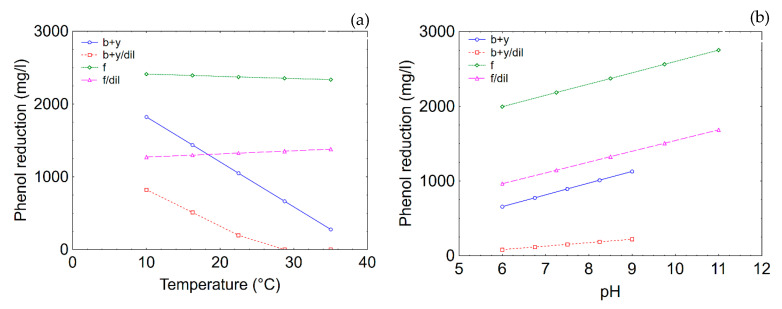
Prediction profiles for the effects of temperature (**a**) and pH (**b**) on phenol reduction. b + y, combination *Bacillus/Candida*; f, fungus (*T. harzianum*); dil, diluted TOPW.

Figure 9 shows the effects of temperature and pH on phenol reduction. The model predicted a strong effect for *Bacillus/Candida*; the effect was different from that recovered for COD. An increase of the temperature, in fact, played a negative role in the reduction of phenols in TOPW (1824 mg/L at 10 °C and 277 mg/L at 35 °C in not diluted TOPW) (Figure 9a). pH was significant for both *T. harzianum* and *Bacillus/Candida,* and the correlation phenol reduction vs pH was positive; that is, an increase of pH caused an increase in the performances (from 657 to 1129 mg/L for *Candida/Bacillus* and from 1994 to 2752 mg/L for *T. harzianum* in undiluted TOPW) (Figure 9b).

### 3.5. Effect of Dilution

The last step of this research was to focus on the effect of dilution on the performances of *T. harzianum* and *Bacillus/Candida*; this variable was studied alone, without using it as an independent variable for the DoE approach, because the stronger effect of pH and temperature exerted a confounding effect.

**Figure 10:**
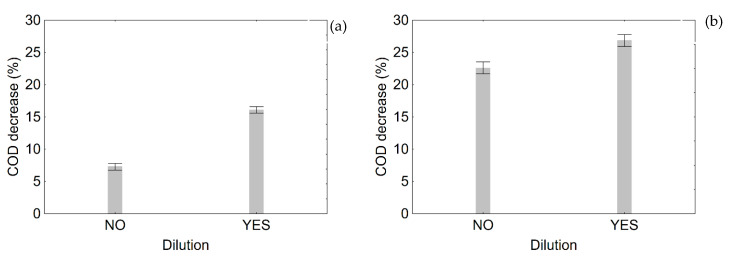
Effect of dilution on the performances of *Bacillus/Candida* (**a**) and *T. harzianum* (**b**) on the reduction of COD. Decomposition of the statistical hypothesis. Bars denote 95% confidence intervals.

Moreover, data were preliminary standardized and reported as performances; the results are shown in Figure 10 as a decomposition of the statistical hypothesis. This figure does not show actual trends, but it only points out the global effect of the factor ‘dilution’ after deleting the effect of the other predictors (pH, temperature and duration). When performing a multivariate analysis of variance, you have some independent variables (in this case pH, temperature, duration and dilution); each factor could be significant or not. A second output is the decomposition of the statistical hypothesis, which shows only the effect for the analyzed parameter. The other parameters are mathematically excluded. Therefore, the meaning of this figure is to show a general effect without a focus on the other variables (for example, duration).

Dilution exerted a significant effect on COD on both *Bacillus/Candida* and *T. harzianum*; the decomposition of the statistical hypothesis suggests a positive effect of dilution on the performance, which increased from 7 to 16% for *Bacillus/Candida* and from 23 to 27% for *T. harzianum*.

## 4. Discussion

In Mediterranean countries, TOPW is often disposed of untreated in land, rivers or the sea, while the most used treatment is disposal in ponds for a forced evaporation, thus leading to the production of malodorous gases by putrefactive or methanogenic bacteria under uncontrolled environmental conditions [1]. Biological technologies (bioremediation and bio-valorization) are promising strategies; they employ microorganisms capable of degrading or converting the pollutants into value-added compounds via specific metabolic pathways. The most efficient approach is anaerobic digestion through methanogenic bacteria [1]. However, there are some limiting factors, such as extreme pH values, high salinity, the unbalanced composition of nutrients and the presence of antimicrobial compounds. Therefore, some authors have proposed a pre-treatment to reduce the pH and/or remove phenols, i.e., fungal metabolization, and the combination of anaerobic treatments with an aerobic step [2].

Generally, fungi are more effective than bacteria at degrading both simple and complex phenols, but some strains of *Bacillus* were reported to effectively reduce phenol content [11]; therefore, a bacterial strain was used in this research in combination with a yeast to increase its efficiency.

The strains proposed in this work were used elsewhere and showed promising trends; namely, *B. pumilus* strain 13M removed aromatic acids in liquid media by 20% or more [7]. *C. boidinii* was selected from OMWW (olive mill wastewater) because of its ability to reduce phenol content by 2000 mg/L after 2 days [8], while *T. harzianum* was grown on OMWW and selected as a resistant strain [9].

Fungi are effective at TOPW decolorization [12]; however, to the best of our knowledge, most papers focused on *Aspergillus niger* [4,13,14,15]. In the past, *Trichoderma* spp. showed potentialities for the treatment of some wastewaters (cork boiling water, tannery, cassava, olive mill wastewater, phenolic resin) due to its usefulness as a decolorization tool, and its ability to reduce COD and phenols [16,17,18,19,20].

The processing parameters analysed in this research were pH, temperature, dilution and duration of the treatment. Concerning pH and dilution, some authors reported that pH adjustment to 6 and dilution by 50% could be promising ways to overcome the problem of wastewater toxicity [1,14,15,21,22].

MANOVA suggests that dilution acted on the yield for COD, but its effect was mild, with an increase of mean efficiency by 4% for *T. harzianum*; the mean effect was higher for the combination *Bacillus*/*Candida* (9%).

This positive effect of dilution on the yield was attributed by Ayed et al. [15] to a reduction of phenols acting negatively on fungi and bacteria.

The effect of pH was controversial, because it acted differently on COD and phenols; namely, an increase of pH had a negative effect on COD yield, and COD decrease was at its maximum at pH 6. There are few evidences on this topic; however, a similar effect was found by Dyan et al. [23] in a plant for the treatment of slaughterhouse wastewater, due to an effect on the viability of the strains.

On the other hand, an increase of pH had a positive effect on phenols removed by *Trichoderma* and *Bacillus*/*Candida*. For fungi, the oxidation of phenolic compounds via the action of tannase and lignin peroxidase is responsible of TOPW bioremediation; however, the absorption of phenolic compounds into the mycelium could be also possible [1,15]. Papdaki et al. [4] studied the effect of a strain of *A. niger* on TOPW; they found a depletion of tyrosol after 2–6 days, and that the pathway involved the synthesis of secoiridoid produced by oleoside and oleuropein aglycon via the action of fungal β-glucosidase and esterase.

Concerning bacteria, a strain of *B. pumilus* was able to degrade protocatechuic acid and caffeic acid; the effect on tyrosol was lower [11].

For fungi, the effect of pH could be attributed to its action on the stability of some enzymes involved in phenol removal [24]. However, as reported above. absorption was probably involved, because a change in the initial pH could probably result in a different distribution of the charges on the surface of bacteria and fungi.

Generally, temperature had a positive effect on both phenol and COD reduction, for a possible effect on the enzymatic kinetics, but further experiments are required to confirm these preliminary findings, as well as to assess the effect of processing parameters on decolorization.

## 5. Conclusions

This research proposes an approach to study the possibility of a bioremediation of TOPW by an alternative fungus (*T. harzianum*) and a combination yeast/bacterium (*Bacillus*/*Candida*). The most important outputs could be summarized as follows. (i) The dilution of TOPW, proposed in the past as a strategy to increase the yield of bioremediation, had an effect only on COD reduction; however, the effect was mild, at least for *T. harzianum*. (ii) pH acted in a different way on phenol removal and COD reduction; an increase of pH caused a reduction of efficiency for COD, while the effect was positive for phenols. (iii) Generally, *T. harzianum* removed higher amounts of phenols and reduced COD more than the combination *Candida*/*Bacillus*, thus confirming the higher efficiency of filamentous fungi.

*T. harzianum* and *Candida*/*Bacillus* did not cause a complete reduction of COD and phenols (to 0); however, the approach proposed in this research has some benefits: (a) a bioreactor is not required, and the microorganisms can act in the tanks used by producers for the long-term storage of TOPW; (b) although temperature is a significant parameter for COD and phenol reduction, both *T. harzianum* and *Candida*/*Bacillus* act in a wide range of temperatures; therefore, the strict control of this parameter is not compulsory.

In conclusion, this research highlights some key-factors for *T. harzianum* and *Candida*/*Bacillus*, but investigations are required for an effective scaling up of the process (agitation, use of large volumes, oxygen, supplementation of TOPW with nutrients) and for an industrial application. Moreover, this work is a screening study/proof of concept, and suggests that some factors (like dilution) could be excluded, along with the need of a risk–benefit analysis for the optimization of the process: COD and phenols, in fact, are regulated in a different way, and it is not possible to find a combination resulting in the highest reduction of both of them; therefore, at the industrial level, a desirability approach could be advisable by setting a break-point for both of them.

## Figures and Tables

**Table 1:** Combinations of the design for the cocktail *C. boidinii*/*B. pumilus*.

Combinations	Temperature (°C)	pH	Duration (days)
A	10	6	7
B	10	6	14
C	10	9	7
D	10	9	14
E	35	6	7
F	35	6	14
G	35	9	7
H	35	9	14

**Table 2:** Combinations of the design *T. harzianum*.

Combinations	Temperature (°C)	pH	Duration (Days)
A	10	6	14
B	10	6	21
C	10	11	14
D	10	11	21
E	35	6	14
F	35	6	21
G	35	11	14
H	35	11	21

**Table 3:** Effect of pH, temperature and duration, as single or interactive terms, on the reduction of COD and phenols by *Bacillus*/*Candida* and *T. harzianum* in TOPW and diluted TOPW from green olive production. R^2^_ad_, adjusted determination coefficient. SE, standard error.

*Bacillus/Candida*				
	TOPW		Diluted TOPW	
	COD Red.	Phenol Red.	COD Red.	Phenol Red.
Temperature	857.25	−1000.25	700.75	-
pH	-	626.38	−97.75	-
Duration	−371.75	384.50	-	-
Temp × pH	-	−582.87	-	-
Temp × Dur	-	-	152.25	-
pH × dur	-	-	-	-
R^2^_ad_	0.997	0.998	0.999	
SE	20.25	20.38	105.13	
***T. harzianum***				
	**TOPW**		**Diluted TOPW**	
	**COD Red.**	**Phenol Red.**	**COD Red.**	**Phenol Red.**
Temperature	1820.37	-	815.25	491.00
pH	−1869.13	809.06	−1375.25	1645.25
Duration	779.63	-	628	−956.00
Temp × pH	-	-278.06	-	−668.13
Temp × Dur	−329.37	-	-	381.23
pH × dur	305.62	-	-	923.13
R^2^_ad_	0.976	0.825	0.926	0.935
SE	109.23	98.60	121.02	154.09
